# DIGITAL CLOCK DRAWING PERFORMANCE ASSOCIATIONS WITH ALZHEIMER’S DISEASE GENETIC RISK SCORE AND APOE IN OLDER ADULTS

**DOI:** 10.1093/geroni/igad104.2233

**Published:** 2023-12-21

**Authors:** Louisa Thompson, Michael Cummings, Sheina Emrani, David Libon, Alvin Ang, Cody Karjadi, Rhoda Au, Chunyu Liu Chunyu Liu

**Affiliations:** Brown University Medical School, Providence, Rhode Island, United States; Boston University Chobanian & Avedisian School of Medicine, Boston, Massachusetts, United States; Brown University, Providence, Rhode Island, United States; Rowan University, Stratford, New Jersey, United States; Boston University, Boston, Massachusetts, United States; Framingham Heart Study, Framingham, Massachusetts, United States; Boston University Chobian & Avedisian School of Medicine, Boston, Massachusetts, United States; Boston University, Boston, Massachusetts, United States

## Abstract

Alzheimer’s disease (AD) is the leading cause of dementia in older adults, but most people are not diagnosed until clinical signs and symptoms are present. Non-invasive and cost-effective digital biomarkers for AD have the potential to improve early detection. We examined the validity of DCTclockTM (a digitized clock drawing task) as an AD susceptibility biomarker. We used two primary independent variables, Apolipoprotein E (APOE) ɛ4 allele carrier status and polygenic risk score (PRS). We examined APOE and PRS associations with DCTclockTM composite scores as dependent measures. We used existing data from the Framingham Heart Study (FHS), a community-based study with the largest dataset of digital clock drawing data to date. The sample consisted of 2,398 older adults ages 60-94 with DCTclockTM data (mean age of 72.3, 55% female and 92% White). PRS was calculated using 38 variants identified in a recent large genome-wide association study (GWAS) and meta-analysis of late-onset AD (LOAD). Results showed that DCTclockTM performance decreased with advancing age, lower education, and the presence of one or more copies of APOE ε4. Lower DCTclockTM Total Score as well as lower composite scores for Information Processing Speed and Drawing Efficiency were significantly associated with higher PRS levels and more copies of APOE ε4. Associations displayed similar effect sizes in men and women. This is the first study to demonstrate significant differences in clock drawing performance on the basis of APOE status or PRS.

